# Optimization and Tradeoff Analysis for Multiple Configurations of Bio-Energy with Carbon Capture and Storage Systems in Brazilian Sugarcane Ethanol Sector

**DOI:** 10.3390/e26080698

**Published:** 2024-08-17

**Authors:** Bruno Bunya, César A. R. Sotomonte, Alisson Aparecido Vitoriano Julio, João Luiz Junho Pereira, Túlio Augusto Zucareli de Souza, Matheus Brendon Francisco, Christian J. R. Coronado

**Affiliations:** 1 Mechanical Engineering Institute—IEM, Federal University of Itajubá—UNIFEI, Itajubá 37500-903, Brazilcesar.sotomonte@unila.edu.br (C.A.R.S.); matheus_brendon@unifei.edu.br (M.B.F.); christian@unifei.edu.br (C.J.R.C.); 2 Chemical Engineering Institute, Federal University of Latin American Integration—UNILA, Foz do Iguaçu 85870-650, Brazil; 3 Department of Planning, Aalborg University, Rendsburggade 14, 9000 Aalborg, Denmark; 4 Computer Science Division, Aeronautics Institute of Technology—ITA, São José dos Campos 12228-900, Brazil; joaoluizjp@ita.br

**Keywords:** bio-energy, BECCS, multi-objective optimization, sugarcane bagasse

## Abstract

Bio-energy systems with carbon capture and storage (BECCS) will be essential if countries are to meet the gas emission reduction targets established in the 2015 Paris Agreement. This study seeks to carry out a thermodynamic optimization and analysis of a BECCS technology for a typical Brazilian cogeneration plant. To maximize generated net electrical energy (MWe) and carbon dioxide CO_2_ capture (Mt/year), this study evaluated six cogeneration systems integrated with a chemical absorption process using MEA. A key performance indicator (gCO_2_/kWh) was also evaluated. The set of optimal solutions shows that the single regenerator configuration (REG1) resulted in more CO_2_ capture (51.9% of all CO_2_ emissions generated by the plant), penalized by 14.9% in the electrical plant’s efficiency. On the other hand, the reheated configuration with three regenerators (Reheat3) was less power-penalized (7.41%) but had a lower CO_2_ capture rate (36.3%). Results showed that if the CO_2_ capture rates would be higher than 51.9%, the cogeneration system would reach a higher specific emission (gCO_2_/kWh) than the cogeneration base plant without a carbon capture system, which implies that low capture rates (<51%) in the CCS system guarantee an overall net reduction in greenhouse gas emissions in sugarcane plants for power and ethanol production.

## 1. Introduction

According to the report from the Intergovernmental Panel on Climate Change in 2018 [1], humans must reduce anthropogenic CO_2_ emission levels by 45% from 2010 to 2030 and reach zero emissions by 2050 to limit global warming to 1.5 °C. The Paris Agreement from 2015 has set a goal for preventing global temperature increases by 2 °C, relative to pre-industrial levels, and seeks to limit temperature increases to 1.5 °C. In this agreement, Brazil pledged to reduce its GHG (greenhouse gas) emissions by 37% by 2030 and 43% by 2050, relative to 2005. Recently, Brazil reinforced its participation in reducing emissions to zero by 2050 at the 2021 Climate Summit.

Carbon capture and storage (CCS) systems and negative emission technologies (NETs) will be essential in meeting this target [2]. CCS systems are already available in the market; however, they are still expensive [3]. A complete CCS system can constitute 80% of the total cost of a plant, including capture, transportation, and storage [4]. A report released by the Global CCS Institute [5] presented different strategies for mitigating global warming and pointed out that bio-energy with carbon capture and storage (BECCS) technologies are crucial.

BECCS technologies refer to the integration of CCS systems with bioenergy-based systems, including biomass-fueled boilers and furnaces, biogas upgrading facilities, and ethanol plants. Biomass, as a renewable energy source, is considered carbon-neutral throughout its lifecycle [6]. Therefore, BECCS is viewed as the most viable approach for achieving negative emissions. This is especially true when compared to the application of CCS to fossil fuel-based systems, which can transform them into negative emission technologies at a cost of up to USD 1000 per tonne of CO_2_ [7].

The main limitation, and what keeps the BECCS systems away from economic feasibility, is the energy penalty associated with its operation, as well as CO_2_ compression, transportation, and storage processes [8]. Therefore, the tradeoff between energy efficiency and CO_2_ capture is key to assessing the technical and economic feasibility of these systems. Fajardy et al. [9] emphasize that biomass residues are a more attractive option economically, since the energy allocated for planting can also be used for other purposes by diversifying the products’ portfolio, like ethanol production from sugarcane. Sugarcane presents one of the highest efficiencies in converting solar energy into biochemical energy via photosynthesis [10], and it is the main biomass feedstock for energy in Brazil, accounting for 11.7 GW (406 thermoelectric plants) of installed capacity.

In fact, sugarcane represents one of the most important energy sources in the world, being widely used for bioethanol production and presenting a self-sustainable energy processing, often using sugarcane bagasse as a renewable solid fuel to simultaneously produce steam for process, bioethanol, and surplus electricity [11]. Moreover, the sugarcane processing sector is widely used for producing sugar and many other inputs for the food industry [12], and since sugarcane biomass has been also highlighted as a sustainable source of renewable hydrogen [13], its thermal cracking has proven to be a valuable way to obtain this energy vector [14].

Despite being a renewable resource, the sugarcane production chain has various environmental impacts, depending on the agricultural practices employed. These practices need to be properly managed to make sugarcane a more sustainable feedstock. A study focused on South Africa by Pryor et al. [15] showed that green cane harvesting could reduce energy inputs by 4% and greenhouse gas (GHG) emissions by 16%. However, mechanization leads to soil compaction and stool damage, resulting in lower yields and increased energy consumption and GHG emissions. Also, the proper use of sugarcane residues for energy production can increase the process efficiency even further [16].

Based on production records for 36 billion liters of ethanol in 2019, a potential capture of 44.77 tons of CO_2_/year is estimated from the fermentation stage in the ethanol production process. For annual sugarcane production at 665.1 Mt, 246.1 Mt of CO_2_/year can potentially be avoided via BECCS systems [17].

Among the available technologies for CCS systems, post-combustion is the most promising carbon capture method [6], given the relative ease of retrofitting existing thermal plants. In this process, CO_2_ is removed from chemical absorption, which is the most widespread technique, given its technological maturity and potential for short-term applications [18], besides being applicable to sources of CO_2_ between 3 and 20% in the gaseous mixture [19].

In the literature, Dubois and Thomas [20], analyzed three different post-combustion chemical absorption configurations and obtained specific energy consumption at 2.39 GJ/ tCO_2_ in the solvent regeneration for a mixture of MDEA 10% + PZ 30%. Bougie et al. [21] demonstrated that mixtures of MEA with other solvents like glycol monomethyl ether (DEGMEE) increased CO_2_ absorption and reduced energy consumption by up to 78%, compared to traditional MEA at 30%. Li et al. [22] used aqueous ammonia to minimize energy consumption when capturing CO_2_. The results indicated potential reductions of 3.3% in plant energy penalty efficiency compared to conventional MEA. Even though other solvents and mixtures may provide better results from an energy point of view and have high corrosion rates [23], MEA is the most used alternative for removing CO_2_, mostly due to its costs [24].

Post-combustion technology based on MEA was evaluated for a BECCS system placed in the Brazilian sugarcane sector by [25], and although the energy penalty varied from 43% to 52%, investing in a BECCS system was placed as a better investment in comparison to a natural gas-based power plant. BECCS investments would be lower, and negative emissions might be achieved.

Therefore, several works on chemical absorption focus on the performance of pilot plants and models/simulations to find process improvements. In this work, the main objective is to investigate the technical feasibility of BECCS systems for use in the sugar energy sector using carbon capture technologies from chemical absorption under different Rankine cycle configurations. Multi-objective optimization will be performed using the metaheuristic Lichtenberg algorithm based on a thermodynamic cycle developed in the Aspen Plus^®^ V11.

## 2. Bio-Energy with Carbon Capture and Storage—BECCS

### 2.1. Power Generation in the Sugar Energy Sector

The Brazilian sugar alcohol sector, with its varied production range, decades of technical knowhow, and appropriate use of industrial waste, is an essential model of sustainability [26]. Due to its advancements and practical knowledge of various methods, the sugarcane sector has incorporated modern cogeneration systems with reheating and regeneration [27], which provide heat and power for auxiliary equipment and plant utilities, besides surplus electricity, which is sold to the grid. Bagasse-fueled boilers operate at pressures ranging from 22 and 85 bar, with live steam at 320 °C, and they are superheated to 480–520 °C, which is the most common operation carried out with superheat steam at 480 °C and 65 bar. Table 1 summarizes the typical operating parameters for cogeneration plants in the sugar energy sector.

**Figure 1:**
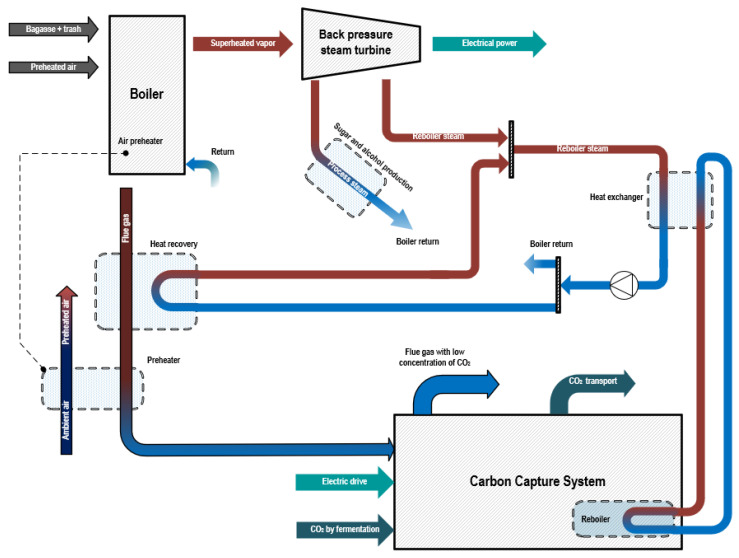
Integrated BECCS system.

Based on the data compiled in Table 1, a plant was studies as a “base case” using typical characteristics for sugar and alcohol plants in Brazil. Table 2 summarizes the data for a sugar mill plant working with 2 Mt of sugarcane per year. The working operation regime was chosen to be 240 days or 5760 h per year, including the harvest and off-season periods, for the running time of the steam cycle, with no modulations to plant operation during the harvest and off-season periods. Figure 1 shows a simplified physical schematic of the proposed BECCS system, with the simulation being carried out using Aspen Plus^®^ V11.

### 2.2. Biomass Combustion

**Figure 2:**
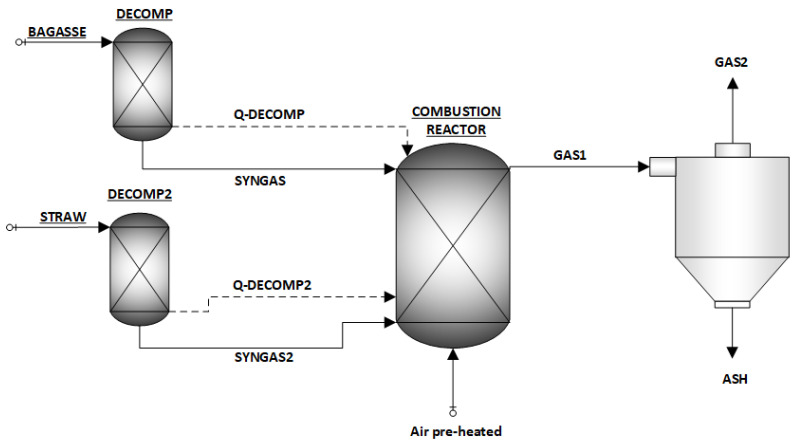
Biomass combustion simulation.

In first stage, bagasse and part of the straw (characterized in Table 3) produced in the field are used as fuel in the boiler. Bagasse with 50% moisture content and straw with 15% moisture content are fed into a yield reactor (RYield) to decompose the solid biomass into its main constituent elements before evaluating the combustion reaction in a Gibbs reactor (RGibbs), disregarding nitrogen oxide formation (Figure 2).

In the Gibbs reactor, the simulation evaluated the biomass combustion reaction in the presence of preheated dry air. The excess air was based on other studies in the literature on bagasse plants from the sector. Carminati et al. [33] used 50% excess air. However, Rayaprolu [34] used a range from 30 to 50% excess air for burning bagasse in more modern boilers. We decided to use the average value of (40%) in the simulation. The combustion exhaust gases pass through a separator to remove ash, leaving only flue gas that is composed of O_2_, N_2_, CO_2_, SO_2_, and H_2_O.

### 2.3. Cogeneration Cycle

**Figure 3:**
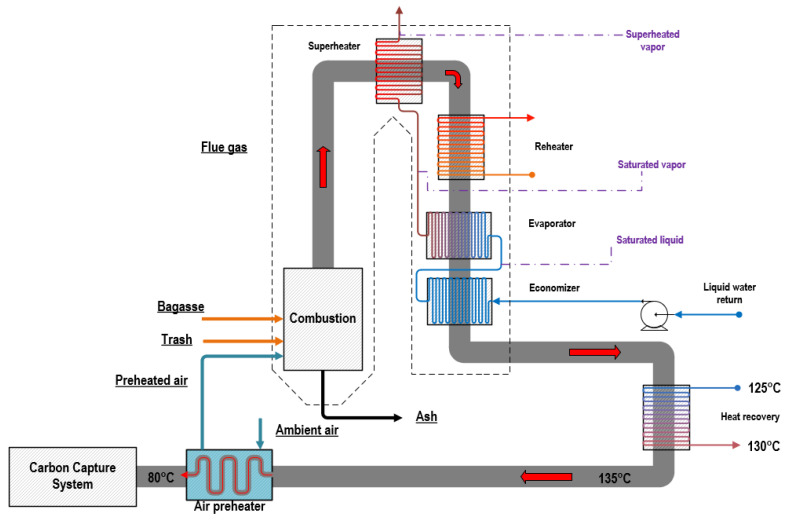
Biomass boiler configuration.

The cogeneration cycle was based on modern cogeneration configurations known in the literature that are used by sugar and alcohol industries. Extractions of steam at 130 °C and 2.5 bar were used to meet plants themal demands, and the operation of the plant was carried out using backpressure turbines, allowing for more heat production downstream. Exhaust gases, which are the products of combustion in the boiler furnace, travel through four primary heat exchanger surfaces in the reheating cycles, namely a superheater, reheater, evaporator, and economizer (Figure 3). An internal heat recovery unit is used to increase the water flow temperature from 125 to 135 °C, which was used to provide heat to the reboiler in the carbon capture and storage (CCS) system. Exhaust gases were cooled to 80 °C by preheating the air used in the combustion simulation.

Six configurations were selected for power generation, as shown in Appendix A. For the simulations, a thermodynamic model based on the Peng Robinson Stryjek–Vera (PRSV) equation of state was used, considering all expansion and compression steps in the turbines and pumps using the isentropic efficiency model, while the heat exchangers were simulated using the on-design model.

The heat supplied in the process and in the reboiler was simulated using a simplified model for heat exchange (cooler). Electrical energy demand for driving motors, lighting, and other auxiliary equipment was taken as being 12 kWh per ton of processed cane. Table 4 summarizes the parameters used in the cogeneration cycle.

### 2.4. Carbon Capture and Storage (CCS) System

Selecting the proper CO_2_ capture technology is directly linked to the combustion or gas formation process. Once separated, CO_2_ has to be compressed and transported in a supercritical state to its final destination. Although it is still considered an expensive technology, capturing CO_2_ via chemical absorption using amine-based solutions is the most dominant technology on the market, and it has been labeled with a Technical Readiness Level of 9 [35]. Moreover, it has been widely studied with a specific focus on the performance of pilot plants and models/simulations for finding process improvements. Table 5 shows the typical carbon capture and storage system’s operating parameters using chemical absorption and MEA as the solvent.

**Figure 4:**
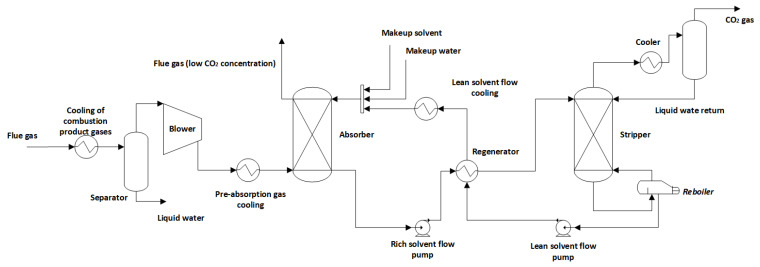
Typical schematic of a chemical absorption system.

To simulate the separation of CO_2_ via chemical absorption in Aspen Plus, the thermodynamic model ElecNRTL (Non-Random Two-Liquid) was used, which is widely used in the literature [43,44]. In Figure 4, a typical flow diagram of a CO_2_ capture system via chemical absorption from the MEA solvent is presented.

At the beginning of the absorption process, exhaust gases leaving the boiler are cooled to 40 °C with the cooler, which is common in absorption columns (no more than 60 °C) to promote CO_2_ absorption using MEA [35]. A separator is used to separate the condensate generated from cooling the gases to ensure there is no liquid in the gases at the blower’s inlet. At the blower, gas pressure is increased (10 kPa) to overcome pressure drops in the absorption column, where gas is placed at the bottom of the column at approximately 50 °C. Both the lean CO_2_ solvent and the makeup flow of water enter the top of the column at 37 °C/1.1 bar. The rich CO_2_ solution is pumped to the stripper column at 2 bar, having passed through the regenerator to be heated to 105 °C; therefore, MEA degradation does not occur [40]. CO_2_ is released from the solution at the top of the stripper, with 99% purity, and it is directed to the compression and transportation stages. At the bottom of the stripper column, the lean CO_2_ solution exits the column at approximately 120 °C and returns to the absorption column, having passed through the regenerator and mixer, where MEA is replenished in the system. All operating parameters in the simulation are summarized in Table 6.

In addition to the CO_2_ produced from the burning biomass, it also accounted the CO_2_ generated from the plant’s fermentation process. Unlike the CO_2_ from exhaust gases, which need absorption systems for separation, the CO_2_ from fermentation can be directly routed to the final transportation system, while considering the electrical power needed to compress it. Table 7 summarizes the correlations for producing CO_2_ from the ethanol production process.

After capture, the CO_2_ must be compressed at high pressures for transportation. The compression process was based on the configurations presented in [40]. Here, CO_2_ was compressed up to 150 bar for transportation. The CO_2_ flows produced by the plant were compressed from 2 to 128 bar using six compression stages, with a compression ratio equal to 2, and intermediate cooling down to 30 °C. After the last compression stage, the CO_2_ was cooled again and compressed to 150 bar and then transported.

## 3. Parametrical Optimization Methodology

**Figure 5:**
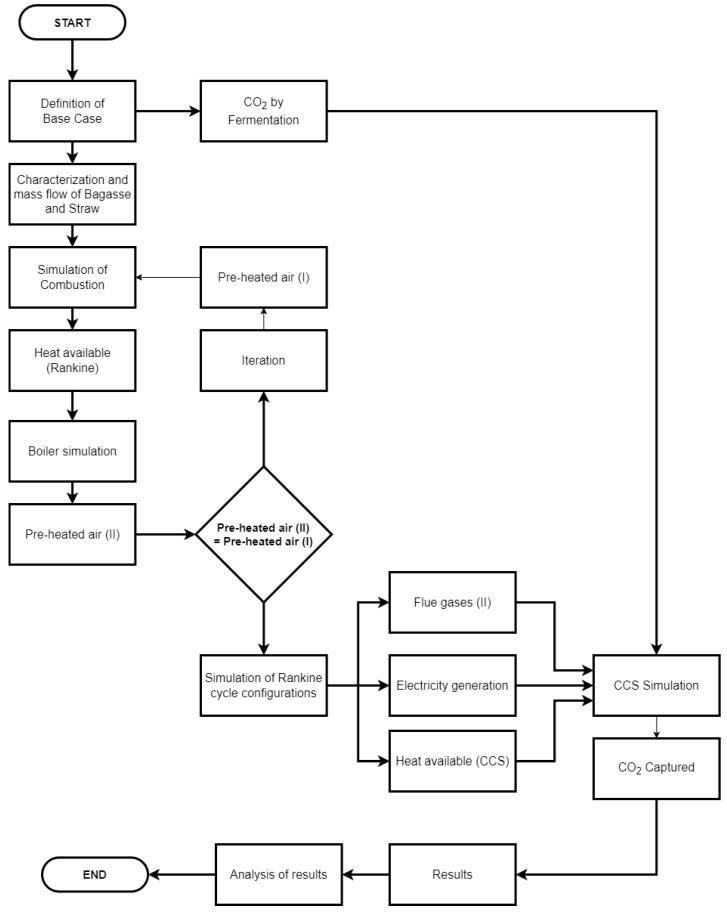
Flowchart of methodology.

The technical and thermodynamic evaluation of the BECCS system involved four stages: (1) simulation of biomass combustion; (2) simulation of the Rankine cycle configurations; (3) simulation of the CCS system; and (4) parametric optimization of the BECCS system. The four steps are shown in the flowchart in Figure 5.

The thermodynamic problem in question can be statistically analyzed using variance analysis. An optimized matrix of experiments can be generated for the problem using the design of experiments. One must define the input variables—which are the variation intervals of each in the thermodynamic cycle—and the response variables.

After analyzing the cycle parameters, a multi-objective optimization can be performed to find the non-dominated solutions to the problem. All non-dominated solutions are optimal, as are those for which it is not possible to improve an objective without negatively affecting another objective. The set of these solutions is called the Pareto front. Meta-heuristics can better handle complex optimization problems where classical methods have limitations, as well as having the ability to handle optimization problems that do not have explicit objective functions. This approach is particularly useful for simultaneously assessing conflicting goals, such as the maximum cogeneration net power and minimum CCS energy penalty.

The Lichtenberg algorithm [46], will be applied for this. This meta-heuristic model was inspired by lightning and Lichtenberg figures, and examples of its application can be found in [47]. Also, the same metaheuristic model has already been validated for other renewable energy systems, such as steam reforming systems [48].

For optimization, one must define the search domain, i.e., the variation ranges for each variable, which are the same as in the design of experiments. So, the parameter optimizer must be adjusted. The following parameters were chosen based on the recommendations from Pereira et al. [46]: Pop = 100; Niter = 100; Rc = 200; Np = 106; S = 1; ref = 0.4; and M = 0.

A relevant indicator of the stripper is the specific consumption of thermal energy per mass of captured CO_2_ (GJ/tCO_2_ or MJ/kgCO_2_), which varies between 3.5 and 7.4 GJ/tCO_2_ (Table 5). It is of global interest that this indicator be as low as possible to reduce the plant’s energy penalty. In this sense, two objective functions were considered in this optimization: maximizing CO_2_ absorption in the CCS system and maximizing the net electrical power ($W_{n}$) (or minimizing the energy penalty). These indicators are the most influential in determining the technical and economic feasibility of BECCS systems.

The following design variables were selected for this study: boiler outlet temperature, reheating temperature, turbine outlet pressure, and the pinch point in the economizer and regenerators. It is important to point out that in order to avoid the algorithm losing much of its efficiency, increasing the total number of simulations, and consuming more computational resources, only the design parameters for the cogeneration cycle were considered in the optimization process. Appendix A summarizes the input variables in the optimization cycle.

## 4. Results

As was mentioned before, the main technical barrier of BECCS systems is the energy penalty associated with CO_2_ capture. For this reason, the reheating cycle with three regenerators was optimized using the net power of the cogeneration cycle as an objective function to evaluate the energy penalty associated with the CO_2_ capture system. The results (Table 8) showed that net electrical power was 62.82 MWe, representing an energy efficiency of 31%, and emissions were equal to 1300 gCO_2_/kWh.

The results show that all the configurations of the thermal system provided a perfect negative correlation between the objective functions for the operating range of the evaluated design variables. For the cogeneration cycles that discarded steam reheating (REG1, REG2, and REG3), the steam temperature at the boiler outlet had the greatest influence on the thermodynamic performance of the system. The higher the temperature of the steam at the turbine input, the greater the enthalpy variation during steam expansion in the equipment. Furthermore, high vaporization temperatures ensured that the steam exiting the last turbine stage remained saturated with pressure parameters close to the lower 250 kPa limit for meeting the plant’s process steam quality conditions.

Raising temperatures close to 520 °C resulted in reduced steam mass flow in the cycle, generating less thermal energy for meeting CO_2_ absorption. A lower temperature at the boiler’s outlet increased steam availability for the processes. Under these operating conditions, higher pressures at the last turbine stage are needed to ensure that steam is saturated for alcohol and CCS production processes, leading to decreased power generation in the cogeneration cycle.

The vaporization pressure and pinch point are important design parameters. Similar to the evaporator temperature, the vaporization pressure is directly proportional to the enthalpy variation during steam expansion in the turbine, promoting power generation. The higher the pinch point, the higher the exhaust gas temperature at the inlet of the heat exchanger, favoring energy generation for the CCS process; however, this decreases the mass flow for the working fluid, decreasing power generation in the power cycle.

For configurations with steam reheating (Reheat1, Reheat2, and Reheat3), the results show that both the vaporization pressure and the low-pressure turbine discharge pressure influenced the thermal system the most, generating more electrical power or heat for downstream plant processes. On the other hand, the vaporization temperature had little influence on the evaluated objectives compared to the configuration without reheating. We also observed that there was a greater interaction between input parameters for the configurations with reheating, although they had little influence on the results when compared to the operating pressures in the cogeneration cycle.

**Figure 6:**
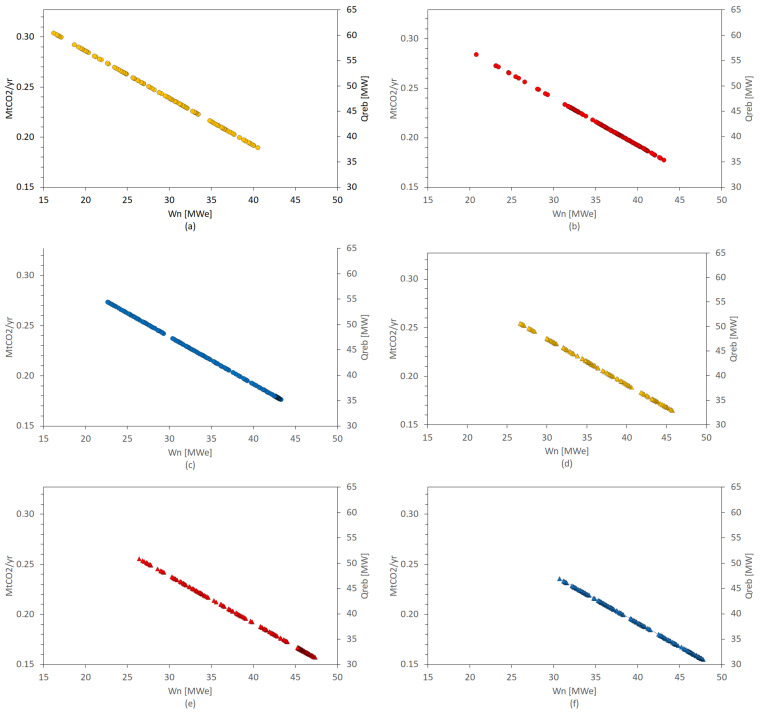
Power generated vs. CO_2_ capture: (**a**) REG1, (**b**) REG2, (**c**) REG3, (**d**) Reheat1, (**e**) Reheat2, and (**f**) Reheat3.

Figure 6 shows the results of the multi-objective optimization for each evaluated configuration. As was mentioned above, we observed that, for each evaluated configuration, there was a negative linear correlation in which an increase in CO_2_ capture capacity led to reduced electrical power generation in the cycle. The heat demand for stripping is critical for releasing CO_2_ from the solvent, enabling its subsequent capture and separation. This demand encompasses sensible heat, which is needed to raise the solution’s temperature; desorption heat, which is responsible for breaking the chemical bonds between CO_2_ and the solution; and latent heat, which is essential for evaporating the solution’s water content. Therefore, a higher CO_2_ capture capacity requires an increased availability of heat in the Rankine cycle for CO_2_ capture purposes. Consequently, a greater capture rate would lead to reduced water vapor available for power generation, resulting in a decrease in power output, commonly referred to as the energy penalty.

Considering CO_2_ absorption, the REG1 configuration was the thermodynamic cycle with the highest heat availability for the CCS system, at approximately 0.304 MtCO_2_/yr, representing a 70.6% CO_2_ capture percentage from nearby exhaust gases; however, it was limited to 16 MWe of net electric power generation (Figure 6a). The Reheat3 configuration was the technological option with the greatest capacity for generating electrical power (47.8 MWe) and for capturing CO_2_, at 0.156 MtCO_2_/yr (Figure 6f). The rest of the configurations showed electricity generation values and CO_2_ capture levels within intermediate ranges between the two previously mentioned configurations.

**Figure 7:**
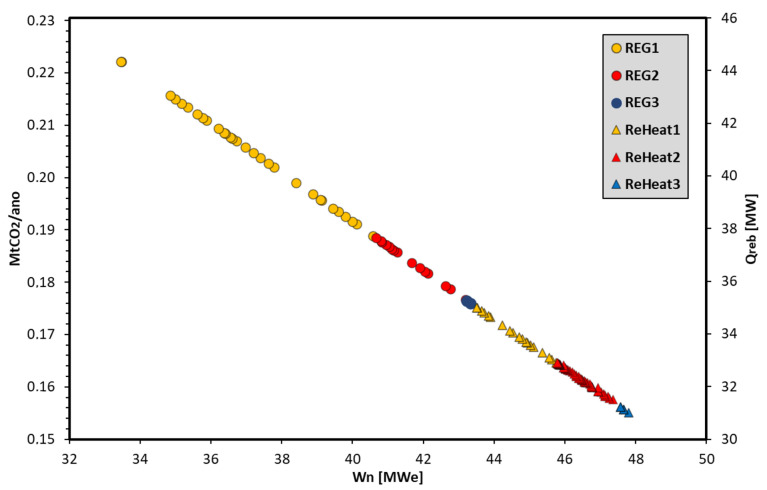
Pareto boundary of the objective functions.

The REG1 configuration is the least complex alternative (fewest devices), i.e., it is the least expensive thermodynamic cycle in terms of installation and maintenance. ReHeat3 is the opposite. Therefore, the more complex cogeneration cycle configurations that produced the same amount of electrical power and captured CO_2_ were excluded from this analysis. Furthermore, the BECCS system showed lower specific emissions relative to the Reheat3 cycle without CCS (1300 gCO_2_/kWh), discarding any set of optimal solutions above this restriction. Figure 7 shows the set of optimal solutions for all evaluated configurations.

The BECCS system was able to obtain a maximum capture of 0.224 Mt/year for REG1 and a CO_2_ capture rate from exhaust gases close to 51.9%; however, it was limited to electrical power generation, which was 33.46 MWe. This was true up to 29.36 MWe, as less than 62.82 MWe was generated by the ReHeat3 configuration without CCS (14.49% penalty on the plant’s electrical efficiency). On the other hand, Reheat3 (with CCS) resulted in more electrical power generation (47.80 MWe) and had a lesser penalty for electrical efficiency (7.41%); however, it had a minimum CO_2_ capture rate of 36.3%, which was emitted by the plant.

**Figure 8:**
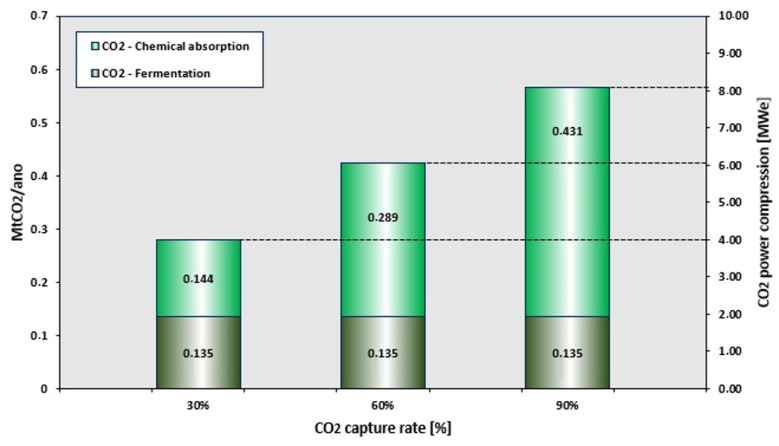
Results for the CO_2_ compression system simulation.

Figure 8 shows three scenarios for percentages of CO_2_ capture, as well as the respective electrical power required for compression at the plant. To compress the CO_2_ generated from fermentation, approximately 2 MWe are needed. Considering the limiting scenario at 90% CO_2_ capture from exhaust gases, 8.24 MWe would be needed to compress the CO_2_ generated by the plant to its maximum capacity, which would be equivalent to 1.48 MWe of power for 0.1 Mt/year of CO_2_ captured at the plant.

**Figure 9:**
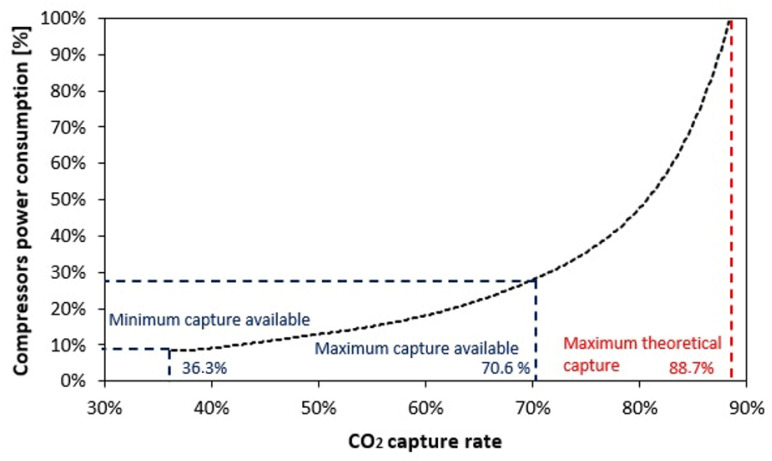
Curve for percentage power consumption of the compressors vs. percentage of CO_2_ capture at the plant.

Figure 9 shows a reduction in the net electric power generated by the BECCS system from the CO_2_ compression system in the plant. The minimum capture point for CO_2_ showed a reduction of 8.27% in the net electric power generated (52.11 to 47.8 MWe), while the maximum capture penalty was 13.67% (38.76 to 33.46 MWe). For a theoretical scenario for a configuration with a greater CO_2_ capture capacity, one could capture up to 88.73% of all the CO_2_ generated at the plant; however, one would need to consume all the electrical power generated to meet the power demands of the compression system.

In this sense, the proposed carbon capture and storage (CCS) approaches have shown the potential to enhance the sustainability of sugarcane-derived bioethanol by further reducing its carbon footprint. Additionally, since CO_2_ can serve as an input for various industrial processes and biofuel production, such as in Fischer–Tropsch synthesis, carbon capture could foster a greater integration between the sugarcane industry and other market sectors, advancing a circular and renewable economy.

## 5. Conclusions

This paper evaluated different bio-energy system configurations integrated with post-combustion chemical absorption (MEA) CO_2_ capture technology. This work differs due to its approach of capturing carbon not only from the CO_2_ of the fermentation process but also from the combustion of bagasse and sugarcane straw, in addition to considering the heat required to supply the ethanol production process in the plant, which globally implies a high thermal demand to be managed from the extractions of steam turbines.

The parametric analyses showed that it is challenging to define the best combination of pressure and temperature parameters, given that the objectives were conflicting (electrical power generation and CO_2_ capture). Furthermore, of the evaluated configurations, different parameters with stronger influences were found for each configuration. Thus, we must use multi-objective and stochastic optimization methods to define the correct operational parameters and tradeoffs between generated electrical power and CO_2_ capture.

From a power generation and carbon capture perspective, the results showed a tradeoff for all the evaluated configurations of the BECCS system. The REG1 configuration resulted in the highest (51.9%) carbon capture with a 14.49% penalty on electrical efficiency (10.49% on the plant’s cogeneration efficiency); therefore, it cannot capture all the CO_2_ generated by the plant (theoretical limitation of 88.7% where all generated electricity would be used to compress the captured CO_2_). The second analysis using (gCO_2_/kWh) indicators showed that CO_2_ capture is more expensive as more power must be used to capture the same amount of CO_2_ in terms of mass, since less electrical power is generated and larger tons of CO_2_ need to be compressed. CO_2_ capture from 51.9% (0.224 Mt/year) would result in emission rates above 1300 g/kWh, which are higher than the plant’s operating emissions with reheating and three regenerators without CCS. On the other hand, the Reheat3 configuration showed the best ratio at 1155 g/kWh (145 g less per kWh generated), with an even smaller penalty on the plant’s electrical efficiency (7.41%); however, it was limited to a minimum capture level of 36.6% for all the CO_2_ emitted at the plant.

The scenarios allowed us to reach reasonable results, where the BECCS system technically partially resulted in negative CO_2_ emissions. This is a plant typical to the Brazilian sugarcane industry, with large demands for suppressed steam from ethanol and sugar production processes (115.5 MW). To capture 90% of all generated CO_2_ from the bagasse and chaff combustion process, 198.4 MW would be needed, and 72% more heat would be destined to a secondary plant process. Future studies are needed to validate the operating ranges and configurations studied in this paper from an economic standpoint.

## Figures and Tables

**Table 1:** Operational parameters of cogeneration plants in the Brazilian sugar energy sector.

	[28]	[27]	[29]
Boiler	22 bar/300 °C	85 bar/520 °C	67 bar/490 °C
	65 bar/480 °C		100 bar/520 °C
	100 bar/530 °C		100 bar/530 °C
Humid Bagasse	50%	50%	50%
Humid Chaff	15%	15%	15%
Humid Fibers	14%	14%	14%
Available Bagasse	280 kg/tc	280 kg/tc	-
Available Chaff	164 kg/tc	164 kg/tc	-
Operation	4464 h	4320 h	4300 h
Milling Capacity	500 tc/h	500 tc/h	465.12 tc/h
Process Steam Consumption	280 kg/tc	450 kg/tc	280 kg/tc
	340 kg/tc		300 kg/tc
	500 kg/tc		500 kg/tc

**Table 2:** Parameters for sugarcane processing.

Annual milling capacity	2,000,000 tc/year
Vapor process consumption	430 kgv/tc
Annual operation time (harvest + between harvest)	5760 h
Cane processing/hour	347.2 tc/h
Bagasse/cane ratio	28%
Annual bagasse production	560,000 tb/year
Available bagasse for producing electricity (90%)	504,000 tb/year
Available bagasse	87.50 tc/h
Chaff/cane ratio	16.4%
Annual chaff production	328,000 tp/year
Available chaff for producing electricity (10%)	32,800 tp/year
Chaff available	1.58 kg/s

**Table 3:** Characterization of sugarcane bagasse and chaff.

Description	Bagasse [a] [b]	Chaff [c]
**Immediate Analysis [% in mass] [a]**		
Humidity	0.0	0.0
Fixed carbon	12.0	7.7
Volatile material	85.0	79.5
Ash	3.0	12.8
**Chemical Analysis [% in mass] [b]**		
Carbon	46.4	49.6
Hydrogen	6.1	6.4
Nitrogen	0.2	0.5
Chlorine	0.0	0.0
Sulfur	0.1	0.1
Oxygen	44.2	30.5
HHV [c]	19.30	20.04

a [30], b [31] and c [32].

**Table 4:** Input parameters for all the evaluated configurations.

Isentropic efficiency of the turbine	85%
Isentropic efficiency of the pump	75%
Inlet temperature for ethanol production	130 °C
Input pressure for the process	2.5 bar
Output temperature for the process	90 °C
Output pressure for the process	1.3 bar
Inlet temperature for the reboiler	130 °C
Inlet pressure for the reboiler	2.5 bar
Output temperature for the reboiler	125 °C

**Table 5:** Operational parameters of cogeneration plants in the Brazilian sugar energy sector.

References	[36]	[37]	[38]	[39]	[40]	[41]	[42]
**Plant/Simulation Absorber**	Plant	Plant	Plant	Plant	Simulation	Plant/Simulation	Plant/Simulation
Combustion gases (Nm^3^/h)	1550	30–110	350	293	368.8 (kg/s)	242–248 (kg/h)	72 (kg/h)
CO_2_ (vol%)	14.2	3–14	15	13.5	13	5.5–9.9	5.4
CO_2_ captured (%)	90	50–75	90	75–89	90	90	75–91
Solvent flow (m^3^/h)	-	30–350	1300	800–1600	740 (kg/h)	-	200 (kg/h)
L/G rate	-	2.8	3.7	3.9–5.8	-	1.7–2.9	-
Temperature (°C)	40	45–50	40	40–60	42	37–40	40
***Stripper***							
Reboiler specific heat duty (GJ/tCO_2_)	3.5	3.98–5.01	3.92	3.77–4.36	3.76	5.2–7.4	5.01
Lean solvent (mol.CO_2_/mol.MEA)	-	0.08–0.09	-	0.28–0.38	0.23	0.17–0.20	0.27
Rich solvent (mol.CO_2_/mol.MEA)	-	0.11–0.14	-	0.46–0.53	0.49	0.38–0.44	0.38
Reboiler temperature (°C)	98–113	120	113.8	105–110	103	108.7–110.4	112.85
Pressure (bar)	1.75–1.90	1–2.5	1.5	1	1.85	-	2

**Table 6:** Operational parameters for the chemical absorption CO_2_ capture system simulated in Aspen Plus.

DATA	
**Model Absorber**	ELECNRTL
Calculation type	Equilibrium
N° of stages	12
Pressure (bar)	1
L/G ratio	3.8
***Stripper***	
Type	Kettle
Reflux rate	0.18
Boilup rate	0.14
N° of stages	20
Reboiler specific heat duty (GJ/tCO_2_)	~3.9
Lean solvent (mol.CO_2_/mol.MEA)	0.19
Rich solvent (mol.CO_2_/mol.MEA)	0.47
Input flow temperature (°C)	105
Operational pressure (bar)	1.8

**Table 7:** Considerations for estimating the CO_2_ captured form the fermentation process.

Parameter	Value	Reference
Ethanol production (L/tc)	86.3	[45]
CO_2_ production per kg of ethanol [kg CO_2_]	0.96	[17]

**Table 8:** Optimal results for the cogeneration cycles.

	ReHeat3 (sem CCS)	REG1	REG2	REG3	ReHeat1	ReHeat2	ReHeat3
W_c_ [MWe]	-	5.30–4.81	4.81–4.63	4.63–4.62	4.62–4.45	4.45–4.34	4.34–4.31
W_n_[MWe]	62.82	33.46–40.58	40.58–43.19	43.19–43.33	43.33–45.75	45.75–47.36	47.36–47.80
*η* [%]	31.01	16.52–20.03	20.03–21.32	21.32–21.39	21.39–22.58	22.58–23.38	23.38–23.60
*η*cog [%]	88.02	77.53–77.05	77.05–78.33	78.33–78.40	78.40–79.60	79.60–80.39	80.39–80.61
CO_2_ [Mt/year]	0.474	0.224–0.190	0.190–0.178	0.178–0.177	0.177–0.166	0.166–0.159	0.159–0.156
CO_2_ capture	0	51.9–44.2	44.2–41.3	41.3–41.1	41.1–38.5	38.5–36.8	36.8–36.3
Emissions [g/kWh]	1300	1300–1215	1215–1190	1190	1190–1169	1169–1155	1155

**Figure A1:**
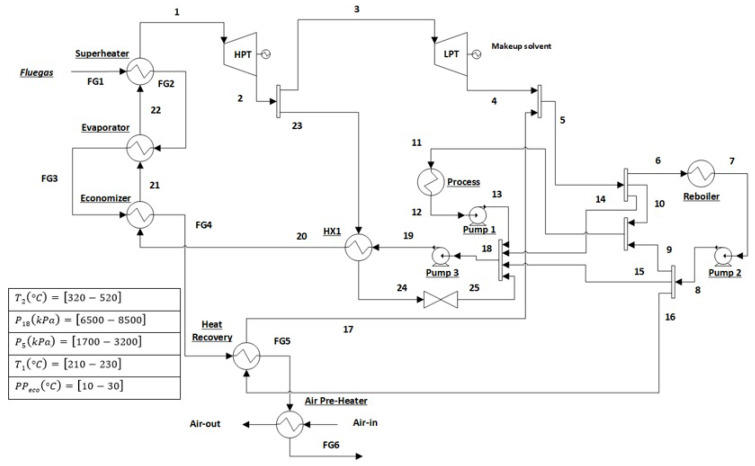
Cogeneration system with regeneration—REG1.

**Figure A2:**
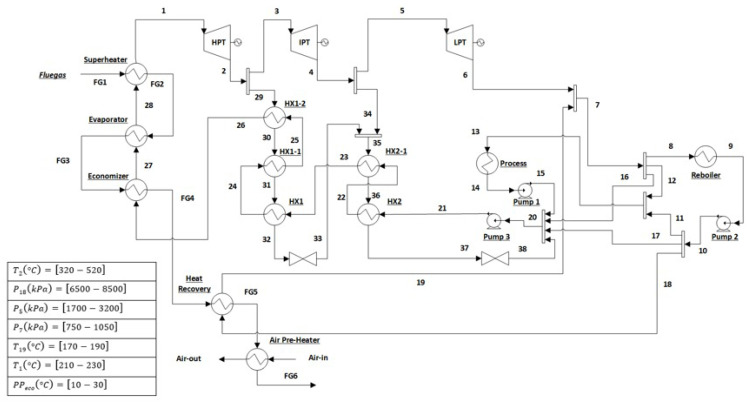
Cogeneration system with regeneration—REG2.

**Figure A3:**
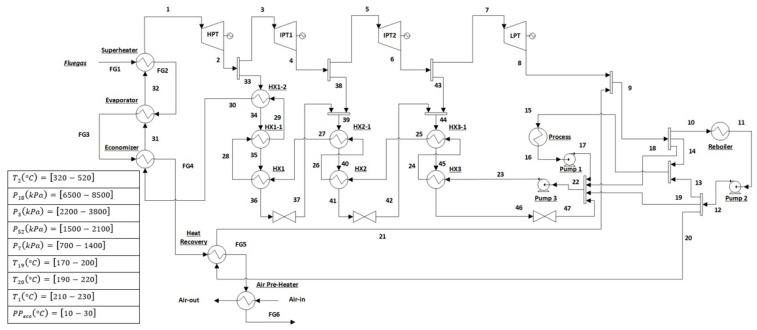
Cogeneration system with regeneration—REG3.

**Figure A4:**
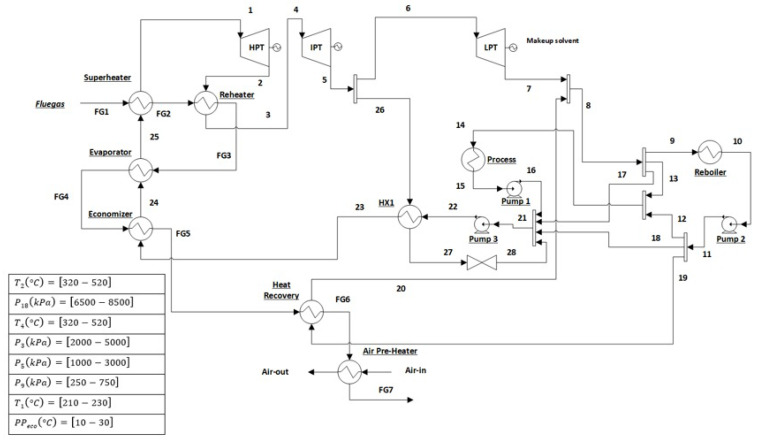
Cogeneration system with reheat and regeneration—Reheat1.

**Figure A5:**
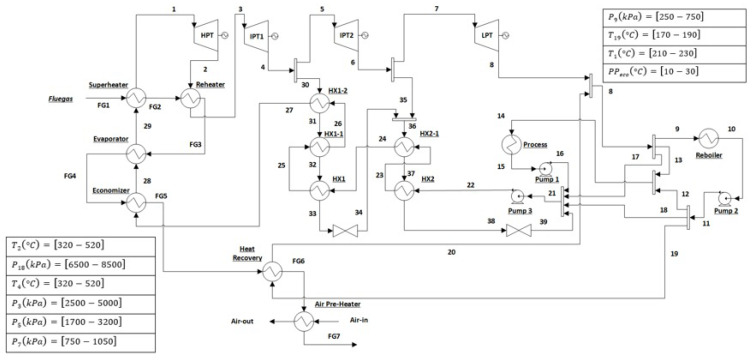
Cogeneration system with reheat and regeneration—Reheat2.

**Figure A6:**
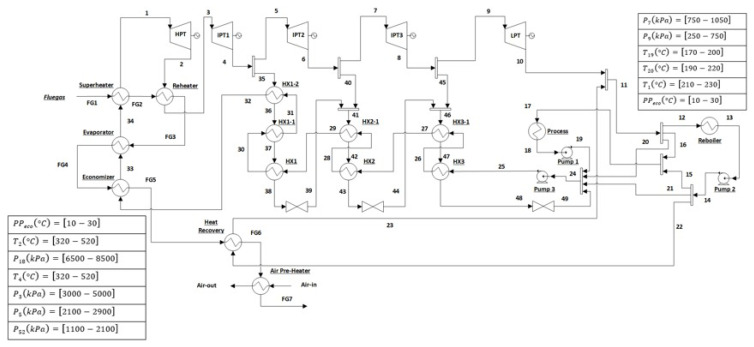
Cogeneration system with reheat and regeneration—Reheat3.

## Data Availability

Data are contained within the article.

## References

[1] IPCC (2018). Global Warming of 1.5 °C. An IPCC Special Report on the Impacts of Global Warming of 1.5 °C above Pre-Industrial Levels and Related Global Greenhouse Gas Emission Pathways, in the Context of Strengthening the Global Response to the Threat of Climate Change.

[2] Intergovernmental Panel on Climate Change (2014). Climate Change 2014 Mitigation of Climate Change.

[3] Wilberforce T., Baroutaji A., Soudan B., Al-Alami A.H., Olabi A.G. (2019). Outlook of carbon capture technology and challenges. Sci. Total Environ..

[4] Blomen E., Hendriks C., Neele F. (2009). Capture technologies: Improvements and promising developments. Energy Procedia.

[5] Global CCS Institute (2019). Bioenergy and Carbon Capture: Perspective.

[6] Emenike O., Michailos S., Finney K.N., Hughes K.J., Ingham D., Pourkashanian M. (2020). Initial techno-economic screening of BECCS technologies in power generation for a range of biomass feedstock. Sustain. Energy Technol. Assessments.

[7] Du Y., Gao T., Rochelle G.T., Bhown A.S. (2021). Zero- and negative-emissions fossil-fired power plants using CO_2_ capture by conventional aqueous amines. Int. J. Greenh. Gas Control.

[8] Julio A.A.V., Escobar Palacio J.C., Rúa Orozco D.J. (2024). Techno-economic and environmental comparison of carbon capture for standalone retrofitting and CO2 hubs in a coal-fueled power complex. Energy Convers. Manag..

[9] Fajardy M., Mac Dowell N. (2018). The energy return on investment of BECCS: Is BECCS a threat to energy security?. Energy Environ. Sci..

[10] Neves M.F., Kalaki R.B. (2020). Bioenergy from Sugarcane.

[11] Lozano M.A., dos Santos R., Santos J.J., Serra L.M. (2024). Optimal modes of operation and product cost allocation in sugarcane steam cogeneration plants. Therm. Sci. Eng. Prog..

[12] Minnu S.N., Bahurudeen A., Athira G. (2021). Comparison of sugarcane bagasse ash with fly ash and slag: An approach towards industrial acceptance of sugar industry waste in cleaner production of cement. J. Clean. Prod..

[13] Julio A.A.V., de Souza T.A.Z., Rocha D.H.D., Rodriguez C.J.C., Palacio J.C.E., Silveira J.L., Muthu S.S. (2022). Environmental Footprints of Hydrogen from Crops. Environmental Footprints of Crops.

[14] de Souza T., Rocha D., Julio A., Coronado C., Silveira J., Silva R., Palacio J. (2021). Exergoenvironmental assessment of hydrogen water footprint via steam reforming in Brazil. J. Clean. Prod..

[15] Pryor S.W., Smithers J., Lyne P., van Antwerpen R. (2017). Impact of agricultural practices on energy use and greenhouse gas emissions for South African sugarcane production. J. Clean. Prod..

[16] Chipfupa U., Tagwi A. (2024). Greenhouse gas emission implications of small-scale sugarcane farmers’ trash management practices: A case for bioenergy production in South Africa. Energy Nexus.

[17] Moreira J.R., Romeiro V., Fuss S., Kraxner F., Pacca S.A. (2016). BECCS potential in Brazil: Achieving negative emissions in ethanol and electricity production based on sugar cane bagasse and other residues. Appl. Energy.

[18] Bhave A., Taylor R.H., Fennell P., Livingston W.R., Shah N., Dowell N.M., Dennis J., Kraft M., Pourkashanian M., Insa M. (2017). Screening and techno-economic assessment of biomass-based power generation with CCS technologies to meet 2050 CO_2_ targets. Appl. Energy.

[19] Julio A.A.V., Castro-Amoedo R., Maréchal F., Martínez-González A., Escobar Palacio J.C. (2023). Exergy and economic analysis of the trade-off for design of post-combustion CO_2_ capture plant by chemical absorption with MEA. Energy.

[20] Dubois L., Thomas D. (2018). Comparison of various configurations of the absorption-regeneration process using different solvents for the post-combustion CO_2_ capture applied to cement plant flue gases. Int. J. Greenh. Gas Control.

[21] Bougie F., Pokras D., Fan X. (2019). Novel non-aqueous MEA solutions for CO_2_ capture. Int. J. Greenh. Gas Control.

[22] Li K., Yu H., Feron P., Tade M., Wardhaugh L. (2015). Technical and Energy Performance of an Advanced, Aqueous Ammonia-Based CO_2_ Capture Technology for a 500 MW Coal-Fired Power Station. Environ. Sci. Technol..

[23] Chao C., Deng Y., Dewil R., Baeyens J., Fan X. (2021). Post-combustion carbon capture. Renew. Sustain. Energy Rev..

[24] Otitoju O., Oko E., Wang M. (2021). Technical and economic performance assessment of post-combustion carbon capture using piperazine for large scale natural gas combined cycle power plants through process simulation. Appl. Energy.

[25] Restrepo-Valencia S., Walter A. (2019). Techno-economic assessment of bio-energy with carbon capture and storage systems in a typical sugarcane mill in Brazil. Energies.

[26] Batlle E.A.O., Julio A.A.V., Santiago Y.C., Palácio J.C.E., Bortoni E.D.C., Nogueira L.A.H., Dias M.V.X., González A.M. (2022). Brazilian integrated oilpalm-sugarcane biorefinery: An energetic, exergetic, economic, and environmental (4E) assessment. Energy Convers. Manag..

[27] Díaz Pérez Á.A., Escobar Palacio J.C., Venturini O.J., Martínez Reyes A.M., Rúa Orozco D.J., Silva Lora E.E., Almazán del Olmo O.A. (2018). Thermodynamic and economic evaluation of reheat and regeneration alternatives in cogeneration systems of the Brazilian sugarcane and alcohol sector. Energy.

[28] Alves M., Ponce G.H., Silva M.A., Ensinas A.V. (2015). Surplus electricity production in sugarcane mills using residual bagasse and straw as fuel. Energy.

[29] Maluf A.B. (2015). Avaliação Termoeconômica da Cogeração no Setor Sucroenergético com o Emprego de Bagaço, Palha, Biogás de Vinhaça Concentrada e Geração na Entressafra. Doctoral Thesis.

[30] Wienese A. Boilers, Boiler Fuel and Boiller Efficiency. Proceedings of the 75th Annual Congress South African Sugar Technologists’ Association.

[31] Turn S.Q., Jenkins B.M., Jakeway L.A., Blevins L.G., Williams R.B., Rubenstein G., Kinoshita C.M. (2006). Test results from sugar cane bagasse and high fiber cane co-fired with fossil fuels. Biomass Bioenergy.

[32] (2001). Evaluation of cyclone gasifier performance for gasification of sugar cane residue—Part 1: Gasification of bagasse. Biomass Bioenergy.

[33] Carminati H.B., Milão R.d.F.D., de Medeiros J.L., Araújo O.d.Q.F. (2019). Bioenergy and full carbon dioxide sinking in sugarcane-biorefinery with post-combustion capture and storage: Techno-economic feasibility. Appl. Energy.

[34] Rayaprolu K. (2009). Boilers for Power and Process.

[35] IEA (2020). Energy Technology Perspectives 2020—Special Report on Carbon Capture Utilisation and Storage.

[36] Moser P., Schmidt S., Sieder G., Garcia H., Stoffregen T. (2011). Performance of MEA in a long-term test at the post-combustion capture pilot plant in Niederaussem. Int. J. Greenh. Gas Control.

[37] Mangalapally H.P., Hasse H. (2011). Pilot plant study of two new solvents for post combustion carbon dioxide capture by reactive absorption and comparison to monoethanolamine. Chem. Eng. Sci..

[38] Kwak N.S., Lee J.H., Lee I.Y., Jang K.R., Shim J.G. (2012). A study of the CO_2_ capture pilot plant by amine absorption. Energy.

[39] Stec M., Tatarczuk A., Więcław-Solny L., Krótki A., Ściążko M., Tokarski S. (2015). Pilot plant results for advanced CO_2_ capture process using amine scrubbing at the Jaworzno II Power Plant in Poland. Fuel.

[40] Farajollahi H., Hossainpour S. (2017). Application of organic Rankine cycle in integration of thermal power plant with post-combustion CO_2_ capture and compression. Energy.

[41] Akram M., Ali U., Best T., Blakey S., Finney K.N., Pourkashanian M. (2016). Performance evaluation of PACT Pilot-plant for CO_2_ capture from gas turbines with Exhaust Gas Recycle. Int. J. Greenh. Gas Control.

[42] Notz R., Mangalapally H.P., Hasse H. (2012). Post combustion CO_2_ capture by reactive absorption: Pilot plant description and results of systematic studies with MEA. Int. J. Greenh. Gas Control.

[43] Morgan J.C., Chinen A.S., Omell B., Bhattacharyya D., Tong C., Miller D.C. (2017). Thermodynamic modeling and uncertainty quantification of CO_2_-loaded aqueous MEA solutions. Chem. Eng. Sci..

[44] Chinen A.S., Morgan J.C., Omell B.P., Bhattacharyya D., Miller D.C. (2016). Dynamic Data Reconciliation and Model Validation of a MEA-Based CO_2_ Capture System using Pilot Plant Data. IFAC-PapersOnLine.

[45] Macedo I.C., Seabra J.E., Silva J.E. (2008). Green house gases emissions in the production and use of ethanol from sugarcane in Brazil: The 2005/2006 averages and a prediction for 2020. Biomass Bioenergy.

[46] Pereira J.L.J., Francisco M.B., Diniz C.A., Antônio Oliver G., Cunha S.S., Gomes G.F. (2021). Lichtenberg algorithm: A novel hybrid physics-based meta-heuristic for global optimization. Expert Syst. Appl..

[47] Pereira J.L.J., Francisco M.B., da Cunha S.S., Gomes G.F. (2021). A powerful Lichtenberg Optimization Algorithm: A damage identification case study. Eng. Appl. Artif. Intell..

[48] de Souza T., Pereira J., Francisco M., Sotomonte C., Ma B., Gomes G., Coronado C. (2023). Multi-objective optimization for methane, glycerol, and ethanol steam reforming using lichtenberg algorithm. Int. J. Green Energy.

